# An immunohistochemical diagnostic panel for Xp11.2 translocation renal cell carcinoma: A diagnostic accuracy study

**DOI:** 10.1097/MD.0000000000050027

**Published:** 2026-07-31

**Authors:** Mengchao Wei, Boju Pan, Wenjie Yang, Weifeng Xu, Jie Dong

**Affiliations:** a Department of Urology, Peking Union Medical College Hospital, Chinese Academy of Medical Sciences and Peking Union Medical College, Wangfujing, Beijing, People’s Republic of China; b Department of Pathology, Peking Union Medical College Hospital, Chinese Academy of Medical Sciences and Peking Union Medical College, Wangfujing, Beijing, People’s Republic of China.

**Keywords:** diagnosis, fluorescence in situ hybridization, immunohistochemistry, translocation renal cell carcinoma

## Abstract

Transcription factor binding to IGHM enhancer 3 (TFE3) break-apart fluorescence in situ hybridization (FISH) is the gold standard for the diagnosis of Xp11.2 translocation renal cell carcinoma (tRCC). However, FISH is costly and not accessible in all laboratories. We aimed to develop a surrogate immunohistochemical panel to recognize Xp11.2 tRCC. We retrospectively enrolled 84 patients who underwent surgery for renal lesions from September 2017 to August 2022. Both immunohistochemistry and FISH analysis were performed on their specimens. The sensitivity, specificity, and accuracy of each immunohistochemical marker and combinatorial immunohistochemical panels were calculated. A total of 17 patients were diagnosed with Xp11.2 tRCC using FISH. In patients with Xp11.2 tRCC, 77% (13/17), 100% (17/17), 88% (15/17), 88% (15/17), 100% (17/17), and 71% (12/17) were tested positive for AE1/AE3, CD10, P504S, PAX8, TFE3, and vimentin, respectively, while 94% (16/17) were tested negative for cytokeratin 7 (CK7). The immunohistochemical panel combining negative CK7, positive CD10, positive P504S, and positive TFE3 exhibited a sensitivity of 0.824, a specificity of 0.672, and a highest accuracy of 0.702. The immunohistochemical panel combining negative CK7, positive CD10, positive P504S, and positive TFE3 was a potentially useful tool for the diagnosis of Xp11.2 tRCC.

## 1. Introduction

Xp11.2 translocation renal cell carcinoma (tRCC) is a rare type of renal cell carcinoma (RCC) characterized by translocations involving chromosome Xp11.2, leading to gene fusions of the transcription factor binding to IGHM enhancer 3 (TFE3) transcription factor gene.^[1]^ The overall incidence rate of Xp11.2 tRCC is relatively low, ranging from 20% to 40% in adolescents and only 1% to 4% in adults.^[2]^ Despite its rarity, this special type of RCC tends to exhibit more aggressive clinical behavior than common RCC subtypes. The optimal therapy for localized Xp11.2 tRCC is surgery, and postoperative adjuvant therapy is highly recommended.^[3]^ For patients with metastatic Xp11.2 tRCC, current treatment options include immunotherapy and targeted therapy.^[4–6]^ Nevertheless, the prognosis of Xp11.2 tRCC is poor irrespective of treatment. The average survival of adult patients with Xp11.2 tRCC is merely 27 months.^[7]^ Therefore, to ensure active treatment, it is critical to diagnose this aggressive type of RCC promptly and accurately following surgical resection.

The clinical manifestation of Xp11.2 tRCC often overlaps with that of other common types of RCC. The majority of patients present with a renal mass revealed by regular physical examination. In terms of histopathological features, the gross findings and microscopic features are not unique enough in Xp11.2 tRCC to help differentiate it from other types of RCC.^[8,9]^ The TFE3 gene is a member of the microphthalmia-associated transcription factor family.^[10,11]^ The rearrangement of the TFE3 gene is crucial in the pathogenesis of Xp11.2 translocation RCC.^[12]^ The TFE3 break-apart fluorescence in situ hybridization (FISH) on formalin-fixed, paraffin-embedded tissue sections is the current gold standard for identifying TFE3 rearrangements and diagnosing Xp11.2 tRCC.^[13–16]^ However, FISH is not universally accessible due to cost and technical requirements. Because TFE3 is the most sensitive and specific marker for Xp11.2 tRCC, the detection of TFE3 using immunohistochemistry was once considered a surrogate for FISH.^[17]^ However, due to differences in fixation times, technical methods, and scoring systems, false-negative and false-positive results of TFE3 immunohistochemistry are frequent. Therefore, the results of TFE3 immunohistochemistry should be cautiously interpreted.^[18,19]^ Regardless of the ambiguous diagnostic efficacy of TFE3 in Xp11.2 tRCC, immunohistochemistry is still a commonly available diagnostic tool. Other immunohistochemical markers, including cytokeratin 7 (CK7), Melan-A, HMB-45, and cathepsin K, are potentially useful for the diagnosis of Xp11.2 tRCC.^[20,21]^ A recent study aimed to establish a panel to differentiate microphthalmia family tRCC from other common renal cell neoplasms.^[22]^ Cathepsin K, CA9, CK7, and parvalbumin were strongly recommended to be incorporated into the final immunohistochemical panel for the diagnosis of microphthalmia family tRCC. However, the diagnostic sensitivity and specificity of the immunohistochemical panel were not mentioned in the study.

To shed further light upon the role of immunohistochemistry in the diagnosis of Xp11.2 tRCC, we investigated the diagnostic efficacy of potential immunohistochemical markers compared with FISH and tried to establish a surrogate immunohistochemical panel for the diagnosis of Xp11.2 tRCC.

## 2. Materials and methods

This study was centrally approved by the Ethics Committee of our hospital in accordance with the Declaration of Helsinki. The approval number was I-22PJ745. Informed consent was waived due to the retrospective design of this study.

### 2.1. Patients

We retrospectively enrolled patients who underwent surgery for renal lesions from September 2017 to August 2022 in the Peking Union Medical College Hospital. The inclusion criteria were as follows: patients were diagnosed as RCC by pathology, and both immunohistochemistry and FISH analysis were performed on their specimens; patients were capable of giving informed consent for the usage of pathological specimens; and patients received no anticancer treatment before surgery. Patients with other malignancies, other renal diseases, severe co-existing morbidities, and organ dysfunction were excluded from enrollment. Age, gender, and laboratory test results, including preoperative white blood cell, platelet, hemoglobin, alanine aminotransferase, albumin, and creatinine, were collected.

### 2.2. Immunohistochemistry

**Figure 1.**
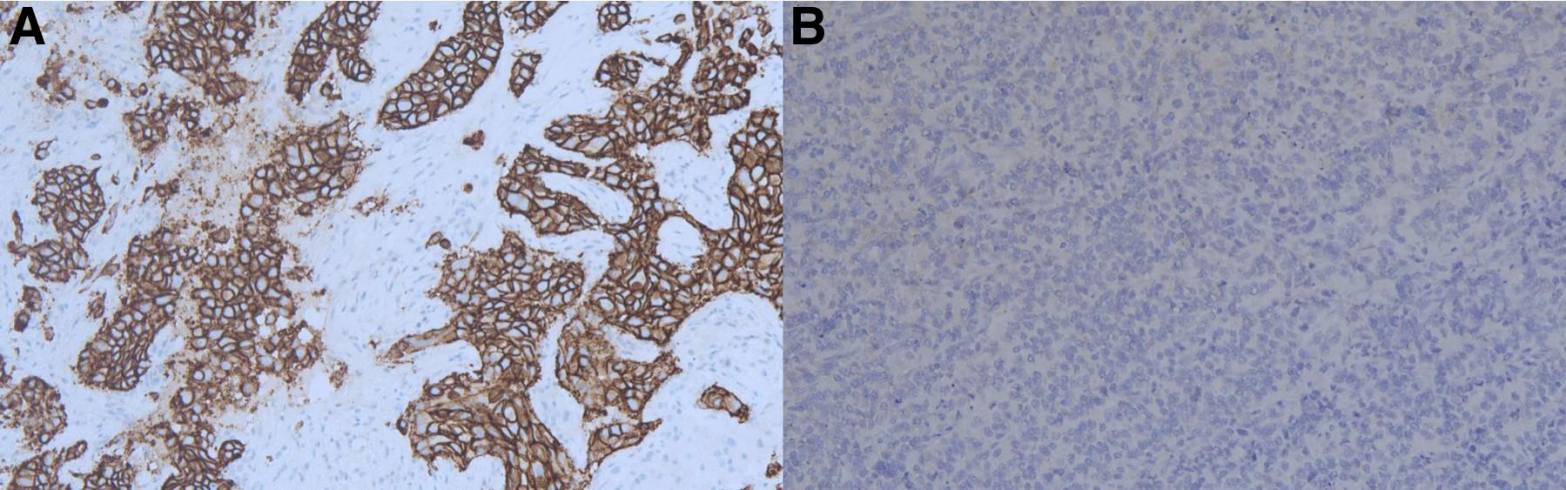
Typical photographs showing positive (A) and negative (B) immunohistochemical results of TFE3 (×40).

Formalin-fixed, paraffin-embedded 4-μm-thick tissue sections of each patient were stained immunohistochemically for the following markers: AE1/AE3 (AE1/AE3), CA9 (multiclone), CD10 (UMAB235), CK7 (UMAB161), EMA (E29), P504S (13H4), PAX8 (OTI6H8), RCC (66.4.C2), TFE3 (EP285), and vimentin (V9). AE1/AE3, CA9, CK7, EMA, TFE3, and vimentin were tested using the DAKO Autostainer Link 48 system (Agilent Technologies). CD10 and RCC were tested using the Leica Bond-III system. P504S and PAX8 were tested using the Ventana BenchMark Ultra system (Ventana Medical Systems, Inc.‌). The immunohistochemistry results were evaluated by 2 independent reviewers, and any disagreements were resolved by consensus review. Cases with >50% of tumor cells showing positive staining were considered “positive,” and cases with <50% were recorded as “negative” (Fig. 1).

### 2.3. Fluorescence in situ hybridization

The FISH assay was performed on unstained slides using a developed and validated commercial break-apart probe purchased from Wuhan Kanglu Biotechnology. The 2- to 4-μm-thick tissue sections from formalin-fixed and paraffin-embedded tissue blocks were immersed in 70% ethanol and then baked at 95°C for 15 minutes after air drying. The slides were incubated in 4 mg/mL pepsin solution at 37°C. The incubation time varied based on the quality of the specimens. A 10-μL TFE3 probe mixture (hybridization buffer: purified H_2_O: probe = 7:2:1) was added to each slide and sealed under an 8 × 8 mm coverslip with rubber cement. The slides were then incubated at 83°C for 8 minutes to co-denature with the probes, followed by hybridization in a humidified chamber at 37°C for 16 hours. After the coverslips were removed, the slides were soon immersed in posthybridization buffer (2 × saline sodium citrate buffer with 0.3% NP40) at 68°C for 2 minutes, followed by a 1-minute wash in purified H_2_O at 37°C. Finally, the slides were air-dried and counterstained with 2.5 μL of 4′,6-diamidino-2-phenylindole II reagent, and 12 × 12 mm coverslips were applied. Fluorescence signals were analyzed using an Olympus BX63 fluorescence microscope. Signals were considered to be split when the green and red signals were separated by a distance of ≥2 signal diameters. One hundred nonoverlapping nuclei were counted for each case. Based on the generally recommended guidelines used by developed TFE3 break-apart FISH assays, a positive result was identified when >10% of the tumor nuclei exhibited the split-signal pattern.^[15,23]^

### 2.4. Statistical analysis

Continuous variables were presented as median and interquartile range. Categorical variables were presented as frequency and percentage. The sensitivity, specificity, and accuracy of each immunohistochemical marker were calculated. To determine the best diagnostic panel for Xp11.2 tRCC, immunohistochemical markers with adequate specificity (>0.7) or sensitivity (>0.7) were tested in a combinatorial manner. All the above results were analyzed using SPSS 19.0 software (SPSS Inc.).

## 3. Results

### 3.1. Patient characteristics

**Figure 2.**
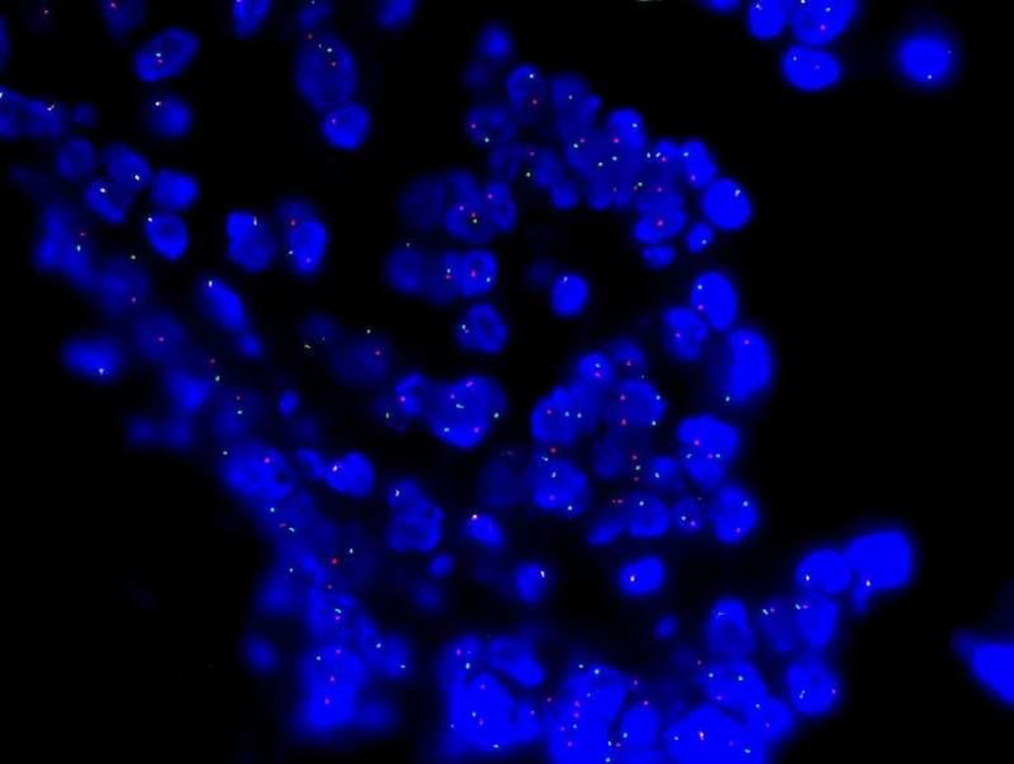
Fluorescence in situ hybridization of Xp11.2 translocation renal cell carcinoma. Distant red and green signals demonstrated the translocation by using a break-apart probe (×1000).

Baseline characteristics are presented in Table 1. After excluding 5 patients with other malignancies and 6 patients with other renal diseases, a total of 84 patients were eventually enrolled in our study, including 54 (64.3%) men and 30 (35.7%) women. The median age was 56 (45, 65) years, and the median tumor size was 3.2 (2.0, 5.4) cm. Following surgery, the specimens of all patients were analyzed using immunohistochemistry and FISH. Finally, 17 patients were diagnosed as Xp11.2 tRCC using FISH. The other 67 patients included 43 clear cell RCCs and 24 papillary RCCs. A typical positive FISH result is shown in Figure 2. In terms of immunohistochemistry, AE1/AE3, CA9, CD10, CK7, EMA, P504S, PAX8, RCC, TFE3, and vimentin were positive in 77 (92%), 49 (58%), 78 (93%), 20 (24%), 57 (68%), 68 (81%), 78 (93%), 39 (46%), 60 (71%), and 73 (87%) cases of all patients, respectively. In patients with negative FISH, 64% (43/67) were tested positive for TFE3.

**Table 1:** Baseline characteristics of the study cohort.

Characteristic	Value
Sex (men/women)	54 (64.3%)/30 (35.7%)
Age, yr	56 (45, 65)
Tumor size, cm	3.2 (2.0, 5.4)
White blood cell, ×10^9^/L	5.8 (5.3, 6.9)
Hemoglobin, g/L	140.5 (125.0, 151.0)
Platelet, ×10^9^/L	222.5 (191.8, 266.5)
Alanine aminotransferase, U/L	17 (13, 23)
Albumin, g/L	42 (40, 45)
Creatinine, μmol/L	71 (60, 87)

### 3.2. Sensitivity, specificity, and accuracy of immunohistochemical markers

Using FISH as the gold standard for the diagnosis of Xp11.2 tRCC, the diagnostic efficacy of each immunohistochemical marker was evaluated. Table 2 presents the sensitivity, specificity, and accuracy of each immunohistochemical marker. CD10 and TFE3 demonstrated a highest sensitivity of 1.000. CK7 demonstrated a highest specificity of 0.716 and a highest accuracy of 0.583. Immunohistochemical markers with adequate specificity (>0.7) or sensitivity (>0.7) were AE1/AE3, CD10, CK7, P504S, PAX8, TFE3, and vimentin.

**Table 2:** Sensitivity, specificity, and accuracy of immunohistochemical markers.

Immunohistochemical marker	Sensitivity	Specificity	Accuracy
AE1/AE3	0.765	0.045	0.190
CA9	0.118	0.299	0.262
CD10	1.000	0.090	0.274
CK7	0.059	0.716	0.583
EMA	0.176	0.194	0.190
P504S	0.882	0.209	0.345
PAX8	0.882	0.060	0.226
RCC	0.588	0.567	0.571
TFE3	1.000	0.358	0.488
Vimentin	0.706	0.090	0.214

CK7 = cytokeratin 7, RCC = renal cell carcinoma.

### 3.3. Immunohistochemical panel

AE1/AE3, CD10, CK7, P504S, PAX8, TFE3, and vimentin were tested in a combinatorial manner to determine the best immunohistochemical panel for the diagnosis of Xp11.2 tRCC. In patients with positive FISH, 77% (13/17), 100% (17/17), 88% (15/17), 88% (15/17), 100% (17/17), and 71% (12/17) were tested positive for AE1/AE3, CD10, P504S, PAX8, TFE3, and vimentin, respectively, while 94% (16/17) were tested negative for CK7. Therefore, different tested panels were combined based on positive AE1/AE3, CD10, P504S, PAX8, TFE3, vimentin, and negative CK7. Because CK7 was the only immunohistochemical marker with adequate specificity, CK7 was considered an essential component of the tested panels. The diagnostic efficacy of these tested panels is presented in Table 3. A total of 63 panels were tested. Negative CK7 combined with positive CD10, P504S, and TFE3 (panel A) showed a sensitivity of 0.824, a specificity of 0.672, and an accuracy of 0.702. The addition of positive PAX8 to the panel (panel B) increased the specificity and lowered the sensitivity, leading to no improvement in the accuracy. To further illustrate the diagnostic efficacy of the 2 panels, we calculated their positive predictive values. The positive predictive value of panel A was 0.389, and that of panel B was 0.382. Therefore, panel A (negative CK7 combined with positive CD10, P504S, and TFE3) was recommended as a surrogate immunohistochemical panel for the diagnosis of Xp11.2 tRCC.

**Table 3:** The diagnostic efficacy of tested panels for the diagnosis of Xp11.2 tRCC.

Panels	Sensitivity	Specificity	Accuracy
Negative CK7+ Positive AE1/AE3	0.765	0.328	0.417
Negative CK7+ Positive CD10	0.941	0.328	0.452
Negative CK7+ Positive P504S	0.824	0.418	0.500
Negative CK7+ Positive PAX8	0.824	0.343	0.440
Negative CK7+ Positive TFE3	0.941	0.507	0.595
Negative CK7+ Positive vimentin	0.647	0.343	0.405
Negative CK7+ Positive AE1/AE3+ Positive CD10	0.765	0.358	0.440
Negative CK7+ Positive AE1/AE3+ Positive P504S	0.647	0.448	0.488
Negative CK7+ Positive AE1/AE3+ Positive PAX8	0.647	0.358	0.417
Negative CK7+ Positive AE1/AE3+ Positive TFE3	0.765	0.552	0.595
Negative CK7+ Positive AE1/AE3+ Positive vimentin	0.588	0.373	0.417
Negative CK7+ Positive CD10+ Positive P504S	0.824	0.463	0.536
Negative CK7+ Positive CD10+ Positive PAX8	0.824	0.373	0.464
Negative CK7+ Positive CD10+ Positive TFE3	0.941	0.552	0.630
Negative CK7+ Positive CD10+ Positive vimentin	0.647	0.373	0.429
Negative CK7+ Positive P504S+ Positive PAX8	0.765	0.448	0.512
Negative CK7+ Positive P504S+ Positive TFE3	0.824	0.418	0.500
Negative CK7+ Positive P504S+ Positive vimentin	0.529	0.478	0.488
Negative CK7+ Positive PAX8+ Positive TFE3	0.824	0.552	0.607
Negative CK7+ Positive PAX8+ Positive vimentin	0.588	0.388	0.429
Negative CK7+ Positive TFE3+ Positive vimentin	0.647	0.567	0.583
Negative CK7+ Positive AE1/AE3+ Positive CD10+ Positive P504S	0.647	0.478	0.512
Negative CK7+ Positive AE1/AE3+ Positive CD10+ Positive PAX8	0.647	0.388	0.440
Negative CK7+ Positive AE1/AE3+ Positive CD10+ Positive TFE3	0.765	0.582	0.619
Negative CK7+ Positive AE1/AE3+ Positive CD10+ Positive vimentin	0.588	0.403	0.440
Negative CK7+ Positive AE1/AE3+ Positive P504S+ Positive PAX8	0.588	0.463	0.488
Negative CK7+ Positive AE1/AE3+ Positive P504S+ Positive TFE3	0.647	0.657	0.655
Negative CK7+ Positive AE1/AE3+ Positive P504S+ Positive vimentin	0.471	0.493	0.488
Negative CK7+ Positive AE1/AE3+ Positive PAX8+ Positive TFE3	0.647	0.567	0.583
Negative CK7+ Positive AE1/AE3+ Positive PAX8+ Positive vimentin	0.529	0.403	0.429
Negative CK7+ Positive AE1/AE3+ Positive TFE3+ Positive vimentin	0.588	0.597	0.595
Negative CK7+ Positive CD10+ Positive P504S+ Positive PAX8	0.765	0.478	0.536
Negative CK7+ Positive CD10+ Positive P504S+ Positive TFE3	0.824	0.672	0.702
Negative CK7+ Positive CD10+ Positive P504S+ Positive vimentin	0.529	0.507	0.512
Negative CK7+ Positive CD10+ Positive PAX8+ Positive TFE3	0.824	0.582	0.631
Negative CK7+ Positive CD10+ Positive PAX8+ Positive vimentin	0.588	0.418	0.452
Negative CK7+ Positive CD10+ Positive TFE3+ Positive vimentin	0.647	0.597	0.607
Negative CK7+ Positive P504S+ Positive PAX8+ Positive TFE3	0.765	0.657	0.679
Negative CK7+ Positive P504S+ Positive PAX8+ Positive vimentin	0.529	0.493	0.500
Negative CK7+ Positive P504S+ Positive TFE3+ Positive vimentin	0.529	0.687	0.655
Negative CK7+ Positive PAX8+ Positive TFE3+ Positive vimentin	0.588	0.597	0.595
Negative CK7+ Positive AE1/AE3+ Positive CD10+ Positive P504S+ Positive PAX8	0.588	0.493	0.512
Negative CK7+ Positive AE1/AE3+ Positive CD10+ Positive P504S+ Positive TFE3	0.647	0.687	0.679
Negative CK7+ Positive AE1/AE3+ Positive CD10+ Positive P504S+ Positive vimentin	0.471	0.522	0.512
Negative CK7+ Positive AE1/AE3+ Positive CD10+ Positive PAX8+ Positive TFE3	0.647	0.597	0.607
Negative CK7+ Positive AE1/AE3+ Positive CD10+ Positive PAX8+ Positive vimentin	0.529	0.433	0.452
Negative CK7+ Positive AE1/AE3+ Positive CD10+ Positive TFE3+ Positive vimentin	0.588	0.627	0.619
Negative CK7+ Positive AE1/AE3+ Positive P504S+ Positive PAX8+ Positive TFE3	0.588	0.672	0.655
Negative CK7+ Positive AE1/AE3+ Positive P504S+ Positive PAX8+ Positive vimentin	0.471	0.507	0.500
Negative CK7+ Positive AE1/AE3+ Positive P504S+ Positive TFE3+ Positive vimentin	0.471	0.701	0.655
Negative CK7+ Positive AE1/AE3+ Positive PAX8+ Positive TFE3+ Positive vimentin	0.529	0.612	0.595
Negative CK7+ Positive CD10+ Positive P504S+ Positive PAX8+ Positive TFE3	0.765	0.687	0.702
Negative CK7+ Positive CD10+ Positive P504S+ Positive PAX8+ Positive vimentin	0.529	0.522	0.524
Negative CK7+ Positive CD10+ Positive P504S+ Positive TFE3+ Positive vimentin	0.529	0.718	0.679
Negative CK7+ Positive CD10+ Positive PAX8+ Positive TFE3+ Positive vimentin	0.588	0.627	0.619
Negative CK7+ Positive P504S+ Positive PAX8+ Positive TFE3+ Positive vimentin	0.529	0.701	0.667
Negative CK7+ Positive AE1/AE3+ Positive CD10+ Positive P504S+ Positive PAX8 Positive TFE3	0.588	0.701	0.679
Negative CK7+ Positive AE1/AE3+ Positive CD10+ Positive P504S+ Positive PAX8 Positive vimentin	0.471	0.537	0.524
Negative CK7+ Positive AE1/AE3+ Positive CD10+ Positive P504S+ Positive TFE3 Positive vimentin	0.471	0.731	0.679
Negative CK7+ Positive AE1/AE3+ Positive CD10+ Positive PAX8+ Positive TFE3 Positive vimentin	0.529	0.642	0.619
Negative CK7+ Positive AE1/AE3+ Positive P504S+ Positive PAX8+ Positive TFE3 Positive vimentin	0.471	0.716	0.667
Negative CK7+ Positive CD10+ Positive P504S+ Positive PAX8+ Positive TFE3 Positive vimentin	0.529	0.731	0.690
Negative CK7+ Positive AE1/AE3 Positive CD10+ Positive P504S+ Positive PAX8+ Positive TFE3 Positive vimentin	0.471	0.746	0.690

CK7 = cytokeratin 7.

## 4. Discussion

As a rare type of RCC, Xp11.2 tRCC is characterized by its aggressiveness and poor prognosis. Careful monitoring and even postoperative adjuvant therapy are highly recommended. Hence, accurate diagnosis of Xp11.2 tRCC is necessary to allow timely intervention following surgical resection.

Argani et al^[17]^ first reported the utility of TFE3 in the diagnosis of Xp11.2 tRCC, with a sensitivity of 97.5% and a specificity of 99.6%, establishing the primary role of TFE3 in this context. The sensitivity was similar at 100% in our study. However, the specificity in our study was only 35.8%, much lower than 99.6%. The corresponding specificity was 57% to 95% in Sharain et al’s study.^[19]^ The inconsistency of specificity may be attributed to several factors. First, the large proportion of tumors not known or suspected to harbor TFE3 gene fusions (screen cases) in Argani et al’s study may exaggerate the specificity result. Second, the technical variations may significantly influence the TFE3 immunostains, with automated assays having greater sensitivity and lesser specificity than manual overnight incubation.^[24]^ Finally, TFE3 has been reported positive in many RCCs without TFE3 rearrangements, such as in 0.4% of clear cell RCC cases,^[25]^ indicating the limited value of TFE3 immunohistochemistry in the differentiation of Xp11.2 tRCC from other common types of RCC.^[26,27]^ Therefore, TFE3 alone is inadequate to diagnose Xp11.2 tRCC accurately.

Despite the unreliability of TFE3 immunohistochemistry in the diagnosis of Xp11.2 tRCC, immunohistochemistry is a familiar and indispensable tool for pathologists because FISH is limited in most laboratories. Several studies have demonstrated the immunohistochemical profile to assist in the classification of TFE3/TFEB-rearranged RCC.^[20,25,28]^ Recently, Caliò et al performed a detailed immunohistochemical analysis of TFE3-rearranged RCC and found that cathepsin K, CA9, CK7, and parvalbumin would help differentiate Xp11.2 tRCC from other common types of RCC.^[22]^ In this study, the diagnostic value of each immunohistochemical marker was evaluated by comparing categorical data for each marker between Xp11.2 tRCC and other common renal cell neoplasms, instead of traditional metrics such as sensitivity, specificity, and accuracy. Our present study also sought to establish a diagnostic panel for Xp11.2 tRCC, but we selected immunohistochemical markers based on the sensitivity, specificity, and accuracy of each tested panel. This methodological difference prevents a direct comparison of panel performance between the 2 studies. Caliò et al’s study investigated both TFE3- and TFEB-rearranged RCC, while our study focused only on TFE3-rearranged RCC, which may account for the variation in the markers included in the final panels. Regarding the immunohistochemical markers that were incorporated into the final panel, TFE3 was an essential element, indicating its significant role in the diagnosis of Xp11.2 tRCC. Compared with TFE3 alone, the panel provided a 0.214 improvement in diagnostic accuracy for Xp11.2 tRCC. CK7 was commonly stained in papillary and chromophobe RCC and was not expressed in TFE3-rearranged RCC.^[22,29]^ Although CD10 was not useful in the differential diagnosis when dealing with TFE3-rearranged RCC in Caliò et al’s study, positive CD10 was observed in 60% cases of Xp11.2 tRCC in previous research.^[30]^ In terms of P504S, it was not tested in Caliò et al’s study; however, it was positive in 95% cases of Xp11.2 tRCC in the previous research.^[25]^

In contrast to previous studies investigating potentially useful immunohistochemical markers for the diagnosis of Xp11.2 tRCC, our study first reported the sensitivity, specificity, and accuracy of the diagnostic panel. The panel achieved an adequate accuracy of 0.702. Given the cost and lack of accessibility of FISH, the panel may serve as a practical surrogate for identifying Xp11.2 tRCC. While FISH is a valuable diagnostic tool for many conditions, its accessibility remains uneven across regions, particularly in resource-limited areas, and its expense can restrict routine use in diagnosing this rare carcinoma. Therefore, our study is meaningful on this point. There is a chance that the panel we recommended could help improve the diagnostic capability of Xp11.2 tRCC in hospitals where FISH is unavailable and provide an alternative diagnostic tool when FISH is not accepted. However, there are several limitations in our study as well. First, our study is single-center and retrospective, and the sample size was relatively small. Large prospective multicenter studies are needed to provide more solid evidence on this issue. Second, given the differences in our institution’s customary testing methods and standard operating procedures, some potentially useful immunohistochemical markers were not included in our study, such as GATA3, parvalbumin, cathepsin K, and HMB-45. This may compromise the comprehensiveness and accuracy of our panel to some degree because cathepsin K and parvalbumin were highly recommended in Caliò et al’s study. A more accurate diagnostic panel deriving from adequate immunohistochemical markers could be explored in the future. Third, although the accuracy of the diagnostic panel is modest, the positive predictive value is as low as 0.389, which may reduce clinical confidence in positive results. However, it would be better to identify more possible positive cases rather than miss 1 real positive case because Xp11.2 tRCC is an aggressive type of tumor.

In summary, the immunohistochemical panel combining negative CK7, positive CD10, positive P504S, and positive TFE3 was a potentially useful tool for the diagnosis of Xp11.2 tRCC. This panel is not a replacement for FISH but a surrogate when FISH is unavailable. Besides, given the low positive predictive value, the panel is useful for screening or preliminary identification, not definitive diagnosis. Large prospective multicenter studies are needed to validate the accuracy of our panel or develop even more accurate diagnostic tools.

## Author contributions

**Conceptualization:** Mengchao Wei, Jie Dong.

**Formal analysis:** Mengchao Wei.

**Project administration:** Mengchao Wei.

**Data curation:** Boju Pan.

**Investigation:** Boju Pan.

**Methodology:** Boju Pan.

**Software:** Wenjie Yang.

**Visualization:** Wenjie Yang.

**Resources:** Weifeng Xu.

**Supervision:** Weifeng Xu.

**Validation:** Weifeng Xu.

**Writing – original draft:** Mengchao Wei.

**Writing – review & editing:** Jie Dong.
